# Crosstalk between protein post-translational modifications and phase separation

**DOI:** 10.1186/s12964-023-01380-1

**Published:** 2024-02-12

**Authors:** Yang Liu, Wenjuan Feng, Yunshan Wang, Bin Wu

**Affiliations:** 1 Jinan Central Hospital, Cheeloo College of Medicine, Shandong University, Jinan, China; 2 Department of Reproductive Medicine, Central Hospital Affiliated to Shandong First Medical University, Jinan, China; 3 Basic Medical Research Center, Central Hospital Affiliated to Shandong First Medical University, Jinan, China

**Keywords:** Phase separation, Post-translational modifications, Biomolecular condensates, Neurodegenerative Diseases, Tumors, Viral Infections

## Abstract

The phenomenon of phase separation is quite common in cells, and it is involved in multiple processes of life activities. However, the current research on the correlation between protein modifications and phase separation and the interference with the tendency of phase separation has some limitations. Here we focus on several post-translational modifications of proteins, including protein phosphorylation modification at multiple sites, methylation modification, acetylation modification, ubiquitination modification, SUMOylation modification, etc., which regulate the formation of phase separation and the stability of phase separation structure through multivalent interactions. This regulatory role is closely related to the development of neurodegenerative diseases, tumors, viral infections, and other diseases, and also plays essential functions in environmental stress, DNA damage repair, transcriptional regulation, signal transduction, and cell homeostasis of living organisms, which provides an idea to explore the interaction between novel protein post-translational modifications and phase separation.

Video Abstract

**Supplementary Information:**

The online version contains supplementary material available at 10.1186/s12964-023-01380-1.

## Introduction

 In recent years, the phase separation of biological macromolecules has been recognized as a prevalent biological phenomenon, which plays important functions in development, environmental stress, DNA damage repair, transcriptional regulation, signaling, and cellular homeostasis of living organisms through its physicochemical properties. As research progressed, it was discovered that protein post-translational modifications (PTM) play an important role in forming and regulating phase separation. Collecting, organizing, mining, and analyzing related data have become research hotspots in the physiological process and the occurrence and development of diseases. Here, we introduce the formation of phase separation, the factors affecting it and the regulation of phase separation mediated by PTMs of proteins, summarize the current research results on PTM of proteins related to eukaryotic phase separation, and look forward to the future development direction, and provide reference and clues for further experimental studies to explore the regulation of protein PTMs on phase separation.

## The formation of phase separation and the factors affecting phase separation

### Concept and formation of phase separation

**Fig. 1:**
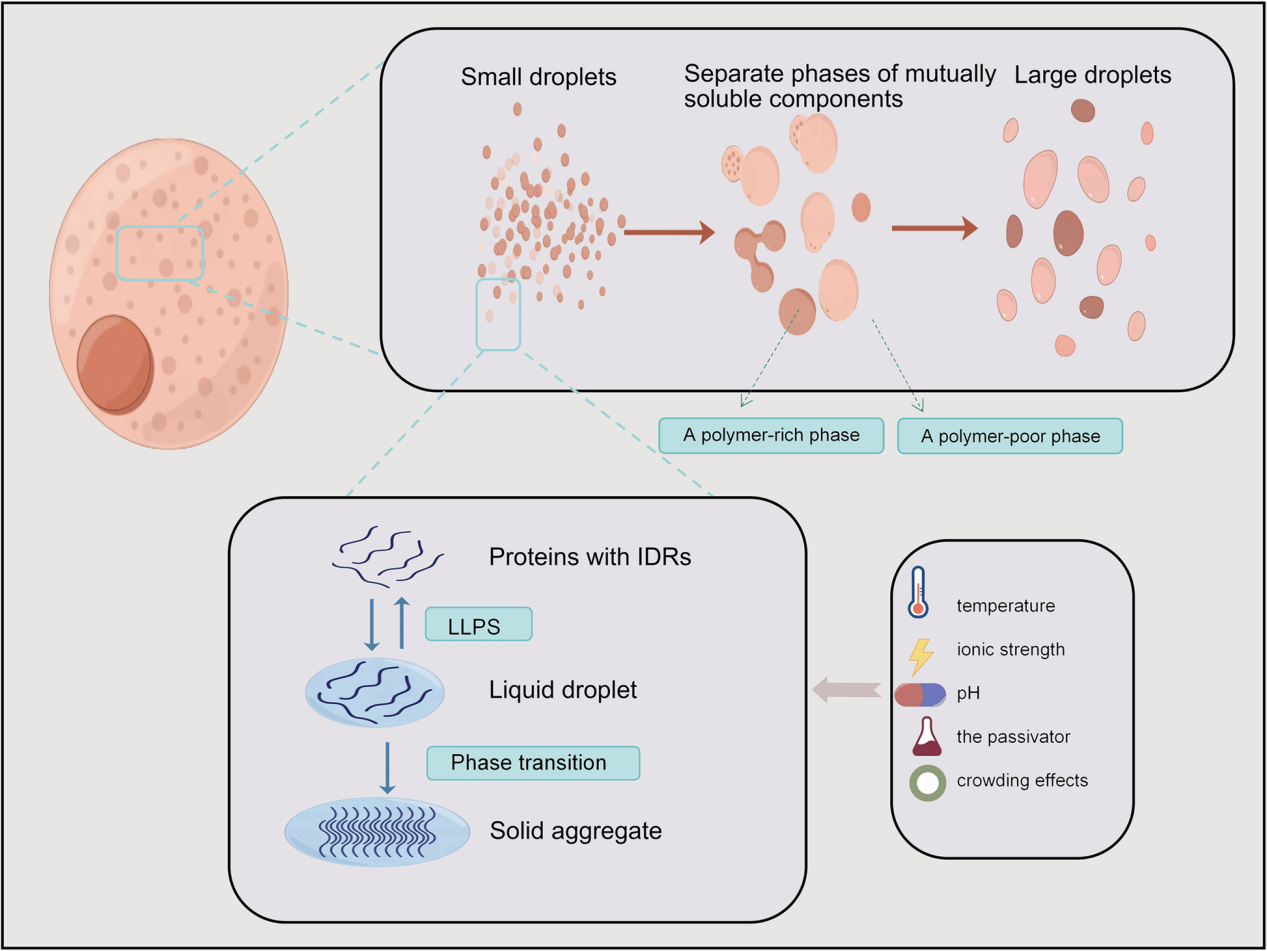
The formation process of LLPS and factors affecting LLPS

 In some external conditions change, some of the molecules in the multi-component system will be enriched to a specific region of the system, the separation of several phases with different components and structures of the process is called phase separation [1]. In 2009, Brangwynne et al. in their study of the mechanisms of fertilized egg polarization and asymmetric division in Caenorhabditis elegans showed that P granules exhibit liquid-like phase change behavior, including fusion, dropping, and wetting [2]. This is the first application of phase separation theory to the field of biology, providing a new perspective for the in-depth study of the physicochemical mechanisms underlying the formation and function of membrane-free functional compartments in cells. Phase separation includes gas-liquid phase separation, [3] liquid-liquid phase separation(LLPS), solid-liquid phase separation, [4] and so on. In biology, LLPS is the most common. LLPS is the creation, under certain conditions, of two separate phases of mutually soluble components in a solution that are constantly exchanged at the phase boundary: a polymer-poor phase and a polymer-rich phase contained therein (Fig. 1) [5, 6]. The condensed phase formed by LLPS helps the complex biochemical reactions within the cell to be able to proceed in an orderly manner in different spaces [7]. The liquid phase state in which intracellular phase separation predominantly exists is usually dominated by surface tension, [8] which gives droplets a spherical shape, small droplets easily fuse to form larger droplets. In certain cases, phase separation within cells can lead to gel-like or even solid-like aggregates (Fig. 1) [9]. Not all intracellular proteins can undergo phase separation; the presence of intrinsically disordered regions (IDR), modular domains, or biomolecules capable of inter- or intra-molecular multivalent interactions with other biomolecules is necessary for phase separation to occur [10–13]. When the molecular solubility in the solution is below the threshold value for the occurrence of phase separation, the molecules can remain dispersed in the solution [8, 14]. When the molecular solubility in the solution exceeds the critical concentration for phase separation, a highly aggregated phase with distinct boundaries and a high degree of motility is precipitated from the solution (Fig. 1) [8, 14].

### Factors affecting the occurrence of phase separation

The most direct factors affecting the occurrence of phase separation depend on the concentration of macromolecules and solutions and their physicochemical properties; [15] In addition, the formation and regulation of phase separation are very sensitive to changes in environmental states, including temperature, ionic strength, pH, as well as the passivator and crowding effects (Fig. 1) [5, 16–19]. For example, the phase separation of the nucleocapsid protein (N-protein) of coronaviruses is an environment-dependent process in which changes in protein concentration, salt ion, and pH concentration can affect the process by which phase separation occurs [20].

Direct adjustment of the critical concentration for phase separation or the concentration of biomolecules in the solution can directly affect the occurrence of phase separation [20]. For example, under low salt conditions in vitro, the short isoform of BRD4 protein (BRD4S) (1-719) formed LLPS droplets at 0.5 µM protein concentration. As the protein concentration of BRD4S(1-719) increased, the number of droplets formed for phase separation and the total area of the droplets increased accordingly, which indicates that the LLPS formed by BRD4S(1-719) under in vitro conditions is concentration dependent [21]. However, under physiological conditions, molecules are not isolated but exist in a crowded complex environment rich in multicomponent abundant proteins, so other competing proteins may interfere with IDR interactions [14]. The lower the critical concentration, the easier it is for the molecules to promote the formation of phase separation through interaction forces [19]. The effect of temperature on phase separation varies depending on the entropy effect system [17]. For phase separation systems with reduced entropy, increasing the temperature promotes phase separation depolymerization, [17, 22] examples include germ granule protein, bovine lens γB-crystallin, and so on [23, 24]. Conversely, for phase separation systems with increased entropy, elevated temperature promotes the formation of phase separation, [17] such as the N-protein of coronaviruses, hydrophobic elastin, and the Alzheimer’s-related protein Tau [20, 25, 26]. Salt ion concentration plays a key role in the formation and regulation of phase separation systems, and increasing ionic strength in phase separation systems where electrostatic interactions dominate weakens electrostatic interactions and is detrimental to the occurrence of phase separation [26]. For example, it has been shown that high salt inhibits the occurrence of tripartite motif-containing protein 21 (TRIM21)-driven LLPS phenomena in which electrostatic interaction forces are the primary mediators of this process [27]. For phase separation systems where hydrophobic interactions dominate increasing the ionic strength reduces the critical concentration at which phase separation of proteins occurs and facilitates the occurrence of phase separation [6, 10]. The effect of pH on phase separation is mainly reflected in the modulation of the occurrence of phase separation by changing the protonation state of amino acids, i.e., by changing the charged properties of amino acids and thus affecting the multivalent intermolecular interactions [25, 28]. In addition to this, the addition of bovine serum albumin (BSA) and protein crowding reagents such as polyethylene glycol (PEG), ficoll, and dextran can mimic the intracellular environment and facilitate the onset of phase separation [29–31]. For example, PEG8000 was added to purified fused in sarcoma (FUS) (1-180) as a macromolecular crowding agent to induce phase separation droplet formation [31].

## PTMs of proteins

### Overview of protein PTMs

Protein PTMs is a covalent modification that occurs in the backbone or side chain of a protein after translation and may alter protein physicochemical properties, spatial conformation, stability, cellular localization, and interactions with other proteins, greatly enriching the diversity of protein structures and functions [32–34]. There are more than 500 known PTMs of proteins, and the common ones include phosphorylation, acetylation, glycosylation, ubiquitination, SUMOylation, and methylation modifications [35]. Protein PTMs are involved in almost all normal life activity processes of cells, greatly enriching the complexity of the organism’s proteome and playing vital biological functions in physiological, pathological, or disease treatment processes. Intrinsically disordered proteins (IDPs) are highly exposed to PTMs as one of the drivers of phase separation [36, 37]. For example, tau is closely associated with a variety of PTMs, such as phosphorylation, ubiquitination, and acetylation. These PTMs enhance or attenuate the LLPS of tau [38–40]. Furthermore, previous studies on N-terminal DDX4 have shown that methylation reduces its LLPS [23].

### Correlation between PTMs and phase separation

PTMs of proteins, including protein phosphorylation modifications, acetylation modifications, methylation modifications, ubiquitination modifications, and SUMO modifications at multiple sites, can affect the charge properties of the modified amino acid residues and charged sequences, change the size of biomolecules, generate new binding sites or change the local structure and conformation, and promote intermolecular multivalent interactions by mediating intermolecular charge-charge, π-π, and cation-π interactions [20, 35, 41, 42], which may regulate the formation of phase separation and the stability of phase separation structures [43].

In addition, phase-separated structures are also involved in regulating protein modifications, such as the cyclin T1 of the positive transcription elongation factor (P-TEFb), which targets RNA polymerase (Pol)II to the phase-separated droplets, and thus contributes to the hyperphosphorylation of the C-terminal domain (CTD) of the RPB1 subunit of human PolII [44]. In addition, it was demonstrated that Arabidopsis blue light receptor CRY2 and transcription factor TCP22 can form a blue light-dependent phase separation, which in turn recruits proteins such as photo regulatory protein kinases (PPKs) into the phase and regulates CCA1 gene expression through various biochemical processes such as protein phosphorylation modification [45]. In 2020, it was demonstrated that the conserved yeast E3 ubiquitin ligase Bre1 binds to the scaffold protein Lge1 and undergoes phase separation through multivalent interactions and that this layered fluid recruits Rad6 and nucleosome substrates to form spatially organized “reaction chambers” that accelerate the ubiquitination of H2B [46]. In 2021, a study found that paraspeckle component 1(PSPC1) can promote the phosphorylation of cyclin-dependent kinase 1 (Cdk1) (Ser-345) by driving the onset of phase separation to recruit the phosphatase serine/threonine-protein phosphatase 5 (PPP5C) in mouse oocyte maturation [47]. In 2022, Dajun Sang et al. demonstrated that MAPK3 and FUS3, as well as Cdk1, are recruited by phase separation into multiple types of synthetic condensates formed by multivalent “scaffolds” in which their phosphorylation is increased [48].

## PTMs of proteins related to eukaryotic phase separation

Based on the excavation of phase separation protein structures and an increasing number of protein modification sites, the linkage between phase separation and protein modification processes has been successively discovered and caused a research boom in the field in the past decade (Table 1). In light of the results of the previous review by Owen I et al., [49] the following section will further review the latest insights on protein PTMs associated with phase separation to help us systematically understand the current common linkage between phase separation and protein PTMs.

**Table 1:** PTMs of proteins regulate LLPS in the physiopathological process

PTM	Protein	PTM Site	Effects of PTM on LLPS (↓↑)	Disease	Physiopathological process	References
	FUS	Ser, Thr	↑↓	ALS, FTD	Disease	[31, 50–52]
	TDP-43	Ser, Thr	↓	ALS, FTD	Disease	[53–55]
	Tau	Ser, Thr	↑↓	Alzheimer’s disease	Disease	[39, 56]
	hnRNPA2	Tyr	↓	ALS, FTD	Disease	[57]
	BRD4		↓	Cancer, AML, etc.	Disease	[21]
	BYSMV P	Tyr	↓		Viral infection	[58]
	SARS-CoV-2 N-proteins	Ser	↑↓	SARS-CoV-2	Viral infection	[59, 60]
	Phosphoprotein	Ser	↑	Measles	Viral infection	[61]
	TAZ		↓		Signal transduction	[62]
	P53	Ser	↓		Genetic transcription	[63]
	NBDY	Thr	↓		Cell function homeostasis	[64]
Phosphorylation	CIRBP	Ser	↓			[65]
	Stargazin	Ser	↑↓			[66]
	PARCL		↓			[67]
	CPEB		↓			[68]
	SMN	Ser	↑	ALS, FTD	Disease	[69]
	HP1α	NTE	↑		Genetic regulation	[42, 70]
	PGL-1/-3	Ser	↑		Environmental stress	[71]
	Liprin-α3	Ser	↑		Genetic regulation	[72]
	FMRP	Ser	↑	Fragile X syndrome	Disease	[73]
	NS2		↑	BT	Viral infection	[74]
	P62	Ser	↑		Disease	[75]
	TIAR-2	Ser	↑		Environmental stress	[76]
	Ddx4	Arg	↓			[23, 77]
	FUS	Arg	↓	ALS, FTD	Disease	[78, 79]
Methylation	hnRNPA2	Arg	↓	ALS, FTD	Disease	[80]
	FMRP	Arg	↓	Fragile X syndrome	Disease	[73]
	LSM4	Arg	↑			[81]
	RAP55A	Arg	↓	Primary biliary cirrhosis	Disease	[82]
	Tau	Lys	↑↓	Alzheimer’s disease	Disease	[38, 83, 84]
	TDP-43	Lys	↑	ALS, FTD	Disease	[85]
Acetylation	DDX3X	Lys	↓	Cancer, Intellectual disability	Disease	[86]
	BRD4	Lys	↑	Cancer, AML, etc.	Genetic transcription;	[21, 87]
	IRF3 / IRF7	Lys	↓		Viral infection	[88]
	FUS	N-terminal	↓	ALS, FTD	Disease	[89]
	H1	Lys			Genetic regulation	[90]
Ubiquitination	Tau	Lys	↑	Alzheimer’s disease	Disease	[91]
	SQSTM1	Leu	↓			[92]
	TRIM		↑		Cell function homeostasis	[93]
SUMOylation	SOP-2	Lys	↑		Genetic transcription	[94]
	SLX4		↑	Fanconi anemia	DNA repair	[95]
	CBX4	Lys	↑	AIDS	Viral infection	[96]
Neddylation	PML/RARα		↓	APL	Disease	[97]
O-GlcNAcylation	GAP	Thr	↓		Signal transduction	[98]
PARylation	CycT1		↓		Genetic transcription	[99]

Arrows (↓): Inhibit LLPS; Arrows (↑): Promote LLPS

### Phosphorylation modifications

**Fig. 2:**
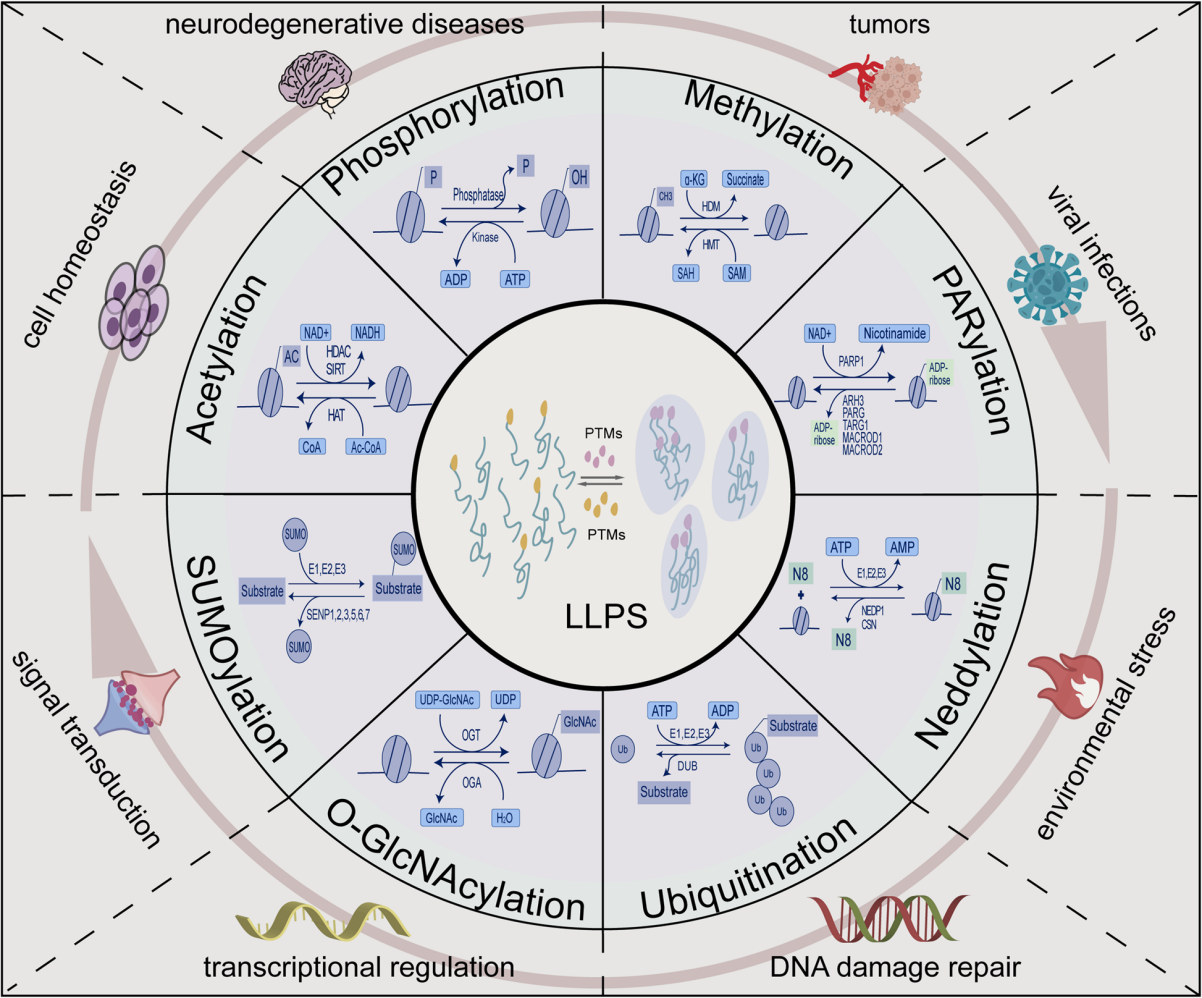
Crosstalk between PTMs and LLPS in the physiopathological process. The large brown semi-circular arrow denotes the illness circle’s ability to rotate, much like a turntable, which we call the “turntable model” here. Crosstalk between PTMs and LLPS has been implicated in numerous areas of illness and biological function, i.e., neurodegenerative diseases, tumors, viral infections, environmental stress, DNA damage repair, transcriptional regulation, signal transduction, and cell homeostasis of living organisms. Furthermore, each PTM is linked to a different disease or biological function, and vice versa

Phosphorylation, one of the most common PTMs of proteins, is an esterification reaction that attaches phosphoryl groups to the hydroxyl groups of the Ser, Thr, and Tyr side chains in proteins and can regulate protein interactions [64, 100]. The effect of phosphorylation on the phase separation of biomolecules is twofold; the addition of phosphate groups can positively or negatively control the formation of phase separation by changing the protein charge [53, 100, 101]. This regulatory role is closely linked to the development of neurodegenerative diseases, [31] tumors, [21] viral infections, [59] and other diseases caused by abnormal phase segregation [73]. In addition to this, it also plays essential functions in environmental stress, [71] DNA damage repair, [50–52] transcriptional regulation, [63] signal transduction, [62] and cell homeostasis of living organisms (Fig. 2) [102].

#### Negative modulation of phase separation by phosphorylation modifications

LLPS has a key role in the development of related neurodegenerative diseases. Given that previous studies noted that phosphorylation of the low-complexity structural domain (LC/LCD) of FUS prevents hydrogel retention [103], Murray DT et al. similarly concluded that phosphorylation of core Ser and Thr residues by DNA-dependent protein kinases prevents binding of FUS-LC hydrogels, leading to phase-separated liquid-like FUS- cleavage of LC droplets [50]. In addition, phosphorylation modifications and phosphomimetic mutations in the FUS low-complexity structural domains were able to reduce the aggregation or liquid-solid transition that occurs in LLPS [101]. However, the phosphomimetic substitution inhibited transient intramolecular and intermolecular LC contacts, disrupting transient intramolecular collapse and intermolecular interactions, providing valuable information for understanding how phosphorylation inhibits LLPS [101]. Ding XF et al. demonstrated that Ser61 site-specific phosphorylation modifications disrupt intramolecular and intermolecular interactions, providing important theoretical support for a role in blocking R2-induced conversion of FUS aggregates to pathological aggregates [31]. Furthermore, phosphorylation of FUS LCD during DNA damage may antagonize FUS phase separation [50–52]. The inhibitory effect of phosphorylation in FUS-LC on FUS LLPS and aggregation is now well known. Notably, the mechanism by which phosphorylation at the atomic level disrupts FUS LLPS and aggregation was first revealed by Lao ZH et al. in 2022 [104].

Some neurodegenerative diseases are associated with abnormal aggregation of TAR DNA binding protein-43 (TDP-43). Phosphomimetic substitutions at Ser48 were found to disrupt TDP-43 polymer assembly [53], and casein kinase 1δ mediated TDP-43 hyperphosphorylation or C-terminal phosphomimetic mutations reduced TDP-43 phase separation and aggregation [54]. Interestingly, Tariq A et al. found that phosphomimetic mutations at the Thr499 and Ser535 positions in the Hsp104 middle domain (MD) enabled Hsp104 to rescue the phase-separation-related aggregation and toxicity of TDP-43, FUS, and α-synuclein in yeast [55]. This regulation could provide a strategy for the treatment of destructive neurodegenerative diseases by designing enhanced Hsp104 disaggregase activity.

Recently phosphorylation has been gradually tapped into regulating the cytoskeleton assembly associated with phase separation. Phosphorylation at Ser262 of the K18 protein in the microtubule-binding domain (MTBD) of tau disrupts binding to microtubulin as assessed by microtubule protein aggregation assay [56]. Furthermore, Savastano A et al. found that LLPS of tau associated with Alzheimer’s disease recruited microtubulin to droplets and promoted microtubule assembly, while phosphorylation at Thr231 disrupted the binding of the proline-rich region P2 of tau to microtubulin and blocked the assembly process [39].

The Ryan VH research team found that tyrosine phosphorylation of the neuropathy-associated heterogeneous nuclear ribonucleoprotein (hnRNPA2) reduced hnRNPA2 phase separation and reduced aggregation of hnRNPA2 disease variants [57].

In recent years, in addition to the numerous studies and research in neurodegenerative diseases, the negative regulatory effects of phosphorylation modifications on phase separation have been gradually uncovered in the following areas. BRD4 protein is closely related to the development of various tumors, and a study in 2020 showed that phosphorylation of p-BRD4S led to a change in the distribution of charge on the surface of protein molecules, which reorganized the structure of protein molecules, thereby inhibiting the formation of LLPS under in vitro conditions and prompting the inactivation of gene transcription, [21] providing new ideas for the development of targeted anticancer drugs. In the context of viral infection, Carlson CR et al. found that unmodified SARS-CoV-2 N-proteins formed ordered gel-like condensates through multivalent RNA-protein and protein-protein interactions, whereas N-protein phosphorylation reduced the interactions and consequently transformed them into more liquid-like droplets, [59] suggesting the effect of phosphorylation on the dual functions of N-proteins in different oligomeric states. Phase segregation of barley yellow striate mosaic virus (BYSMV) phosphoprotein (P) in plants could be inhibited by host CK1-dependent phosphorylation of the intrinsically disordered P region [58]. In terms of signaling, TAZ is an important effector downstream of the Hippo signaling pathway, and its phase separation phenomenon is directly influenced by phosphorylation. When phosphorylated by the Hippo signaling pathway kinase LATS2, the TAZ phase separation phenomenon is inhibited, which prevents the recruitment of transcriptional regulators into the phase change compartment and affects downstream target gene expression. This is the first molecule to elucidate that signaling pathway-specific molecules can signal by way of LLPS, revealing a novel mechanism for phase separation to promote transcription and providing a new direction for the development of disease-therapeutically relevant interventions [62]. In terms of transcriptional regulation, human p53 is a transcription factor that regulates the transcription of multiple target genes. p53 phosphorylation was found by Dai ZJ et al. to promote p53 incorporation into transcriptional complex droplets and inhibit p53-mediated transcriptional droplet aggregation, revealing that p53 phosphorylation alters its LLPS behavior [63]. Lenard AJ et al. found that serine-arginine protein kinase 1 (SRPK1)-mediated phosphorylation of the RG/RGG region of cold-inducible RNA binding protein (CIRBP) impairs LLPS in vitro, which may affect RNA stability and regulate gene expression and cellular function [65]. In the functional homeostasis of cells, the generation of membraneless organelles by LLPS is widely recognized as the mechanism by which many disordered biomolecules within cells can order their physicochemical reactions, which is important for organizing cellular activities. It has been shown that changes in the phosphorylation state of peptides reversibly regulate the liquid organelle model of biomolecules, supporting the key role of phosphorylation modifications in dynamically regulating the liquid intracellular compartments generated by phase separation [102]. For example, LLPS of NBDY microproteins is thought to potentially regulate the formation of membrane-free organelles, the phosphorylation of NBDY drives the dissociation of these droplets [64]. In addition, the negative regulatory effect of phosphorylation on phase separation was confirmed in studies of the Intrinsically disordered plant protein PARCL and the vertebrate CPEB protein [67, 68].

#### Positive regulation of phase separation by phosphorylation modifications

Multiple disease processes were able to discover that phosphorylation modifications can also promote phase separation through intermolecular interactions. In Alzheimer’s studies, it was determined that MARK2 phosphorylation promotes LLPS of the Tau repeat structural domain [25]. Subsequently, by using high molecular weight hyperphosphorylated tau isolated from human Alzheimer’s brain, post-translationally modified recombinant tau was observed to form tau droplets in neurons in vitro, [105] providing strong evidence that phosphorylation promotes phase separation of full-length tau. In a study of the neuromuscular disease spinal muscular atrophy (SMA), Schilling M et al. found that the Ser49 and Ser63 phosphorylation control complexes of the human survival motor neuron protein (SMN) coalesce in Cajal [69]. In a study of fragile X syndrome, TsangB et al. experimentally demonstrated that phosphorylation increased the local negative charge density of fragile X mental retardation protein (FMRP), suggesting that phosphorylation of the low-complexity disordered region at the C-terminus of FMRP may increase its phase separation by increasing the propensity for multivalent electrostatic interactions [73]. The human selective autophagy receptor protein p62 was found to be enriched in cellular inclusion bodies of various human diseases, and phosphorylation of its Ser403 site promotes the binding of p62 protein to ubiquitin chains, thereby facilitating the phase separation of p62 protein to form p62 bodies, [75] providing a theoretical basis for studying the formation of other membrane-free structures in the field of autophagy and the mechanisms of related diseases.

In terms of gene regulation, phosphorylation of the N-terminal extension of human heterochromatin protein 1 (HP1α) associated with heterochromatin gene silencing promotes phase separation formation [70]. Site-specific protein modifications regulate the onset of phase separation, and notably other point mutations do not affect droplet formation, a process confirmed in a study in which Ser760 of Liprin-α3 eliminated Liprin-α3 phosphorylation by a single point mutation [72]. As scientists continue to develop and advance in the field of phase separation mechanisms, in 2022 HerC et al. further demonstrated the importance of electrostatic interactions due to HP1α phosphorylation in LLPS, reporting that phosphorylation redistributes the charge in the protein sequence and enhances the interaction between the NTE and hinge regions. At the same time, it provides an additional negative charge, introduces repulsive forces between other regions of the protein, and leads to protein conformational expansion at high concentrations, among other important mechanisms [42].

Environmental stress-induced changes in phosphorylation also affect the state of phase separation. Under heat stress conditions, mTORC1-mediated phosphorylation of PGL-1/-3 is elevated in P-particle components, and PGL-1/-3 undergoes accelerated phase separation to form PGL particles resistant to autophagic degradation, [71] which can maintain embryonic viability during heat stress, suggesting a protective role of phosphorylation modifications regulating phase separation in the organism under stress conditions. In 2020, Liu ZY’s team demonstrated that stress-induced phosphorylation of Hsp27 impairs the inhibitory activity of Hsp27 in LLPS of FUS-LC to preserve the liquid phase to prevent amyloid fibril formation, [106] providing a deeper understanding and insight into the molecular mechanisms and important roles of membraneless organelles to maintain the liquid or gel phase. In some neuronal injury responses, TIAR-2 can form liquid-like TIAR-2 granules by LLPS and inhibit axonal regeneration. However, the non-phosphorylatable TIAR-2 variant does not form granules and is unable to inhibit axonal regeneration. The critical role of phosphorylation-mediated phase separation in axon regeneration can be seen [76].

In the field of viral infection, measles virus inclusion bodies are formed by a LLPS process and phosphorylation of phosphoproteins Ser86 and Ser151 can improve the efficiency of the inclusion body assembly [61]. PTM is a key step affecting viral inclusion body assembly. In investigating the potential mechanisms of LLPS in virus-infected cells, phosphorylation of its nonstructural protein 2 (NS2) was found to induce and regulate phase separation of NS2 in bluetongue virus, [74] and Ser51 phosphorylation in the N-protein of globally transmitted SARS-CoV-2 was found to potentially inhibit the RNA-binding ability of the NTD structural domain, further promoting phase separation of the N-protein [60].

The effect of phosphorylation at different sites of the same protein on phase separation may be different. For example, phosphorylation of Ser78 of postsynaptic density protein 95 (PSD-95) inhibits phase separation of GluN2B and the auxiliary protein stargazin, whereas phosphorylation of Ser116 induces phase separation of stargazin [66]. Phase separation has been reported to play an important role in the phosphorylation modification of proteins involved in β-catenin, Pol II, PPP5C CHK1, CTD, MAPK3, and Fus3, providing data for insight into the prevalence and biological functions of phase separation [44, 45, 47, 48, 107].

### Methylation modifications

Methylation modification transfers activated methyl groups provided by S-adenosylmethionine (SAM) to specific protein residues by methyltransferases (Fig. 2) [108]. In recent years, a large amount of experimental data has shown that methylation negatively modulates the tendency of phase separation. Nott TJ et al. demonstrated that Ddx4, the major component of Nuage protein, forms structures in vitro that are indistinguishable from intracellular membraneless organelles and form droplets in living cells. However, methylation of arginine in Ddx4 significantly destabilizes droplets and reduces LLPS, [77] which provides ideas for the dynamic regulation of biota compartmentalization. In another related study of membraneless organelles, protein arginine methyltransferase PRMT1 knockdown impairs the localization of RAP55A in the P-body, suggesting that elimination of methylation may have a positive effect on phase separation [82]. In neurodegenerative diseases, FUS arginine methylation has been shown to tend to inhibit phase separation by modifying the interaction of the cation π with tyrosines in the N-terminal LCD, [78, 79] and asymmetric dimethylation in cells decreases nuclear aggregation of FUS [79]. In the same year, a study found that asymmetric dimethylation of hnRNPA2 LCD reduced co-phase separation and co-aggregation with the LCD at the C-terminus of TDP-43 by disrupting arginine-aromatic interactions [80]. In 2020, a study showed that dimethylarginine (DMA) of poly-GR, a dipeptide repeat protein (DPR) associated with TDP-43 inclusions, in the brains of C9orf72 FTD/ALS patients reduced the strength of interactions between poly-GR molecules and decreased the phase separation of poly-GR [109]. Furthermore, in another disease study, Tsang B et al. found that methylation of the low-complexity disordered region (LCR) at the C-terminus of FMRP reduced phase separation in vitro, [73] supporting the role of methylation on the propensity for phase separation. Interestingly, although the positive effect of methylation on phase separation is less reported, it is undeniable that methylation modifications can promote the formation of phase separation [81]. For example, Arribas-Layton M et al. found that symmetric arginine dimethylation of the RGG structural domain of Lsm4 stimulated the accumulation of processing bodies [81].

### Acetylation modification

Acetylation modification is the process of transferring acetyl groups of acetyl coenzyme A to protein amino acid residues in the presence of enzymatic/non-enzymatic action (Fig. 2) [110, 111]. Acetylation modifications are divided into two main categories: those that occur at the lysine end of the protein, and those that occur at the N-terminal end of the protein [110].

A growing number of studies have shown that lysine acetylation modifications of proteins associated with neurodegenerative diseases can mediate the tendency of phase separation and influence the disease process. In 2018, data while studying the aggregation properties of tau showed that hyperacetylation of tau by p300 histone acetyltransferase (HAT) inhibited LLPS and heparin-induced aggregation of tau and prevented microtubule assembly that effectively affected disease progression [38]. Acetylation of residues K321 and K274 of 4R0N tau has been reported to inhibit tau aggregation. Interestingly, [83] acetylation at 4R2N tau residue K274 significantly reduced the critical concentration of tau for LLPS and induced conformational changes in tau, and suggested that acetylation of tau at residue K274 could increase tau aggregation [84]. This suggests that acetylation of the K274 residue produces different aggregation tendencies for different tau isomers. A large amount of TDP-43 can be acetylated, and in 2022 Garcia Morato J et al. found that acetylation of the K136 site of the RNA recognition domain of TDP-43 impaired its RNA binding and splicing ability. This failure of RNA interactions triggered TDP-43 phase separation [85]. Imbalance in the dynamic regulation of lysine acetylation and deacetylation of TDP-43 protein may be closely associated with diseases such as amyotrophic lateral sclerosis (ALS) and frontotemporal lobar degeneration (FTLD) [112]. Acetylation modifications also regulate the assembly of membraneless organelles. For example, Saito M et al. reported that the RNA decarboxylase DDX3X, an important component of stress granules (SGs), is a novel substrate for the deacetylase HDAC6. Acetylation on multiple lysine residues of the N-terminal IDR of DDX3X (IDR1) impairs droplet formation. And the enhancement of LLPS propensity by deacetylation of DDX3X-IDR1 by HDAC6 is necessary for SG maturation [86]. In terms of gene expression, p300 histone acetylation antagonizes chromatin phase separation and reduces the formation of droplets in the nucleus [87]. However, in the presence of polybrominated structural domain proteins, such as BRD4, highly acetylated chromatin forms new phase separation states, and these droplets have different physical properties, and their non-fusion with unmodified chromatin droplets have important implications for our understanding of the organization and regulation of eukaryotic genomes [87]. Subsequently, Han X et al. in their study of the role of BRD4 short isoforms in phase separation and active gene transcription found that BRD4S LLPS is formed through multivalent interactions of IDRs and regulates chromosomal DNA-histone interactions through histone lysine acetylation [21]. In 2022, Qin Z et al. demonstrated that the addition of the acetyl portion at specific lysine residues of the IRF3/IRF7 DNA-binding domain (DBD) eliminates IRF3/IRF7 LLPS, while SIRT1 recognizes acetylated IRF3/IRF7 and promotes LLPS through catalytic deacetylation reactions, elucidating a deacetylation-mediated IRF3/IRF7 LLPS by a novel mechanism of innate antiviral immune control [88]. Currently, there is some progress in protein N-terminal acetylation-mediated phase separation studies, and in 2021 Bock AS et al. observed a difference in the LLPS propensity of N-terminal acetylated FUS-LC compared to those without modification. Furthermore, the team evaluated the aggregation propensity of N-terminal acetylated FUS-LC and found that N-terminal acetylation can slow down aggregation under certain conditions [89].

### Ubiquitination modifications

Ubiquitination is a common PTM in eukaryotic cells, and the covalent attachment of one or more ubiquitin molecules to multiple sites and proteins by the action of ubiquitin-activating enzyme 1 (E1), ubiquitin-conjugating enzyme 2 (E2) and ubiquitin ligase 3 (E3) (Fig. 2) [113]. Deubiquitinase (DUB) plays a role in deubiquitination [114]. In recent years, an increasing number of experimental data have shown crosstalk between PTM and phase separation of proteins.

In 2021 Höllmüller E et al. proposed that site-specific ubiquitination plays a general regulatory role for spliceosomal H1 and that K64 site-specific ubiquitination affects H1-dependent chromosome assembly and phase separation [90]. In 2022 several studies further confirmed that ubiquitination modifications regulate the formation and stabilization of phase separation. For example, Parolini F et al. found that enzymatic ubiquitination of the Alzheimer’s-related protein tau stabilized droplets and prevented aggregation-associated lysis [91]. Gao K et al. described that SPOP binding and induction of nondegradative ubiquitination modification of SQSTM1 at L420 can reduce SQSTM1 body formation, liquid phase condensation, dimerization, and ubiquitin-binding capacity [92]. Furthermore, Tozawa T et al. found that activation of the intracellular ubiquitin cascade reaction promoted the tripartite motif (TRIM) family of ubiquitin ligases molecules into cytoplasmic bodies via LLPS, separating potentially harmful excess TRIM molecules from the cytoplasmic environment [93]. Gao Y et al. found that endoplasmic reticulum-localized AGO proteins recruit LTn1 through lipid-mediated phase separation formation of AGO condensates to catalyze de novo peptide ubiquitination [115, 116].

Phase separation allows ubiquitination and subsequent proteasomal degradation by triggering ubiquitin ligase substrates that activate ubiquitin ligases, which may play a key role in regulating ubiquitin-dependent protein homeostasis [117–119]. In 2018 Bouchard JJ et al. found that substrates trigger phase separation of speckle-type POZ protein (SPOP) in vitro and co-localization in membraneless organelles in cells while cancer-associated mutations in SPOP disrupt mislocalization due to phase separation and thus reduce the level of ubiquitination in cells [117]. The direct role of LLPS in stimulating histone ubiquitination was further elucidated by Gallego LD et al. Lge1, together with Bre1-Rad6 and nucleosomes, forms a spatially organized “reaction chamber” that stimulates H2B ubiquitination [46].

### PTMs of ubiquitin-like proteins

With the discovery of various ubiquitin-like proteins and ubiquitination modifications, the correlation between PTMs of ubiquitin-like proteins and phase separation has been gradually investigated, such as small ubiquitin-related modifier (SUMO), neural precursor cell-expressed developmentally downregulated 8(NEDD8), etc., has been gradually investigated.

#### SUMOylation

SUMO, short for Small Ubiquitin-like Modifier, belongs to the ubiquitin-like protein family. Similar to ubiquitin, SUMO can be covalently attached to lysine side chains in various target proteins. Notably, SUMOylation distinguishes itself from other PTMs by not being a chemical group added to amino acid residues, [89] but rather by forming an isopeptide bond with the target protein. This bond is created through the coordinated activity of specific enzymes, namely the E1 SUMO activation enzyme, E2 SUMO conjugation enzyme, and E3 SUMO ligase (Fig. 2) [113]. Additionally, members of the SENP family possess the ability to cleave the isopeptide bond between SUMO and the target protein [114, 120]. With the continuous research and expansion of the research, the involvement of SUMOylation in the phase separation process is partially understood. It was found that SOP-2, one of the components of Polycomb-related complex 1 (PRC 1), could phase separate and that SUMOylation could regulate this phase separation process, as evidenced by an increase in the diameter and mobility of microdroplets [94]. In acquired immunodeficiency syndrome (AIDS), the phase segregation property of CBX4 allows it to recruit the catalytic subunit EZH2 at the retroviral HIV-1 promoter, which plays a key role in EZH2 SUMOylation and HIV-1 latency [96]. In addition to this, SLX4 can form phase separated droplets within the cell and SUMO-SIM interactions promote the assembly of SLX4 condensates [95].

#### Neddylation

Compared to other ubiquitin-like proteins, the NEDD8 is a molecule with the greatest similarity to ubiquitin, which binds to substrate proteins in a modification process called Neddylation [121, 122]. COP9 signalosome (CSN) and NEDD8 protease 1 (NEDP1), which release substrates and NEDD8 (Fig. 2) [123, 124]. In 2022, A study demonstrated that PML/RARα fusion proteins that drive chromosomal translocation production in acute promyelocytic leukemia (APL) are assembled by LLPS into nucleosomal structures. Further investigation of the mechanism revealed the presence of novel PTMs of ubiquitin-like proteins, Neddylation modification, that disrupt the phase separation process of PML/RARα by facilitating its binding to DNA, ultimately interfering with the assembly of PML nucleosomes [97]. This is the first time that the ubiquitinylation-like modification Neddylation regulates the phase separation process, which further expands the scope of PTMs in the field of phase separation.

### Glycosylation modifications

#### O-GlcNAcylation

GlcNAcylation is linked and involved in regulating the phase separation process (Fig. 2). In 2022, Lv P et al. demonstrated through extensive experimental data that T1306 O-GlcNAc of SynGAP prevents the formation of hydrogen bonds with PSD-95, disrupts protein interactions, and thus inhibits LLPS formation, and that O-GlcNAc-dependent LLPS is subject to O-GlcNAc transferase (OGT) and O-GlcNAcase (OGA) reversible regulation [98]. This finding further opens up a new perspective in the study of the correlation between PTMs and phase separation of proteins and pushes the research process in this field to a new level.

#### Poly ADP-ribosylation (PARylation)

PARylation is an important PTM that involves the addition of poly(ADP-ribose) (PAR) onto specific proteins [125]. This process is primarily catalyzed by a family of enzymes called Poly(ADP-ribose) polymerase (PARP), with PARP1 being the most well-known [125, 126]. PARP1 converts NAD + into ADP-ribose and then adds individual ribose units onto specific amino acid residues of target proteins, forming PAR chains [127]. Hydrolytic enzymes such as PAR glycohydrolase (PARG), ADP-ribosylhydrolase 3 (ARH3), TARG1, MACROD1, MACROD1, etc. can reverse this process (Fig. 2) [128, 129]. Under physiological conditions, PARylation regulates the phase-separation-mediated assembly and disassembly of stress granules containing various RNA-binding proteins (RBPs) [130]. In addition, PARylation modifications and phase separation play an important role in the maintenance of genomic transcriptional stability after DNA damage. In 2022 Fu H et al. revealed that poly(ADP-ribose) polymerase 1 (PARP1)-mediated CDK9-cyclin T1 (CycT1) PARylation through the PARylation-dependent suppression of CycT1 phase separation and hyperphosphorylation of Pol II CTD by P-TEFb inhibits Pol II elongation and induces global transcriptional shutdown in response to DNA damage [99]. Additionally, it was shown that PARylation levels are a major regulator of the assembly-disassembly kinetics of ribonucleoprotein (RNP) granules (e.g. hnRNP A1 and TDP-43) containing disease-associated RBPs [131].

### Hydroxylation modifications

There is no systematic study and interpretation of LLPS related to hydroxylation modifications. However, the possibility that JMJD6 catalysis of lysine hydroxylation regulates higher-order protein associations through effects on LLPS and the actions of this process on membraneless organelles or cellular condensates was proposed in 2022 based on the results of the study [132]. This suggests a new way of thinking about the further development of our field in the future.

### Other novel protein modifications

The research on novel protein modifications such as sulfation, acylation, glutamylation, palmitoylation, carbonylation, glutarylation, myristoylation, crotonylation, malonylation, succinylation, etc. is still in the initial stage and relatively few, the crosstalk of these novel protein modifications and phase separation need to be further explored.

## Issues and prospects

This paper provides an overview of the interactions between PTMs of various proteins and the phenomenon of cytosolic phase separation. It reviews current research on the intricate links between PTMs and cytosolic phase separation. In addition, the paper provides the latest knowledge on intracellular activities and macromolecular assembly, while extensively discussing the topic of the formation of membrane-free compartments and how they depend on PTMs. The understanding of the spatiotemporal dynamics of physiological responses regulated by PTMs at the subcellular level is deepened.

The current understanding of the crosstalk between phase separation and PTMs is mostly confined to the description of the phenomenon, and there is still uncertainty about the causal relationship between them and the specific regulatory mechanisms [133, 134]. Therefore, digging deeper into the biomolecular motifs where phase separation occurs, exploring the interactions between multiple modification sites of phase separation-associated proteins and phase separation, and determining the regulatory relationships and biological functions are our current urgent problems [135]. Pathological aggregation of proteins is closely related to the occurrence of diseases, such as tau [136]. In the future biological field, there is a great deal of potential for exploring how to utilize PTMs on the regulatory mechanism of phase separation to understand the pathogenesis of diseases and to develop targeted drugs [135–138].

## Data Availability

Data sharing does not apply to this article as no datasets were generated or analyzed during the current study.
